# Risk factors and self-management predictors of activities of daily living in patients with heart failure: A 12-month prospective cohort study

**DOI:** 10.1097/MD.0000000000049195

**Published:** 2026-06-05

**Authors:** Meng Ning, Zhiyuan Li, Chong Zhang, Yingwu Liu, Chenguang Zheng

**Affiliations:** a Central Hospital, Tianjin University (Tianjin Third Central Hospital), Tianjin, China; b Neuroscience Intensive Care Unit, The Second Affiliated Hospital of Zhejiang University School of Medicine, Hangzhou, China; c Medical College, Tianjin University, Tianjin, China.

**Keywords:** activities of daily living, diabetes mellitus, heart failure, self-management, valvular heart disease

## Abstract

Impaired activities of daily living (ADL) significantly affect the prognosis and quality of life in patients with heart failure (HF). We aimed to identify the key clinical and self-management predictors of ADL 12 months after hospital discharge. This prospective cohort study enrolled 162 hospitalized patients with HF stratified into low-ADL (ADL < 100, n = 66) and high-ADL (ADL = 100, n = 96) groups based on 12 months of follow-up. The baseline characteristics, comorbidities, biomarkers (B-type natriuretic peptide [BNP]), and self-management domains (psychological, drug, dietary, and symptom management) were compared. Logistic regression was used to identify ADL predictors. Subgroup analyses of left ventricular ejection fraction (LVEF) were performed. The low-ADL group had significantly higher rates of valvular heart disease (VHD; 12% vs 3%, *P *= .025) and diabetes mellitus (29% vs 16%, *P* = .043), elevated BNP levels (median 474.9 vs 398.0 pg/mL, *P *= .039), and poorer self-management scores (*P *< .05). Multivariable analysis confirmed that diabetes (adjusted odds ratio [aOR], 0.33; 95% confidence interval [CI], 0.14–0.80; *P* = .014) and VHD (aOR, 0.19; 95% CI, 0.04–0.80; *P *= .024) were independent negative predictors. Symptom management was the strongest positive predictor (crude OR, 12.71; 95% CI, 3.38–47.74; *P *< .001; aOR, 6.26; 95% CI, 1.44–27.19; *P *= .014). Stratification by LVEF revealed that diabetes mellitus disproportionately impaired ADL in patients with heart failure with reduced ejection fraction (HFrEF; defined as LVEF < 50%; OR, 0.21; 95% CI, 0.06–0.82). The factors influencing ADL scores changed over the 12-month follow-up period. Diabetes, VHD, and poor symptom management were key predictors of long-term ADL impairment in patients with HF. Targeted interventions addressing symptom management and comorbidity control, particularly in patients with HFrEF, may improve functional outcomes.

## 1. Introduction

Heart failure (HF) is a major global health challenge affecting over 64 million individuals worldwide. It is associated with significant morbidity, mortality, and health care burden. Among the various patient-centered outcomes, the ability to perform activities of daily living (ADL) is a critical determinant of the quality of life, independence, and risk of hospital readmission in patients with HF.^[1,2]^ The reduced ability to perform ADL reflects the functional impact of the disease and predicts a poorer long-term prognosis, including increased mortality and reduced survival time.

Although numerous studies have established clinical and biochemical predictors of traditional HF outcomes, such as mortality and hospitalization, the specific factors associated with long-term functional capacity, particularly ADL, remain understudied.^[3]^ This knowledge gap is noteworthy because ADL preservation is fundamental to maintaining patient autonomy and mitigating caregiver burden. HF pathophysiology, often compounded by multiple comorbidities, directly and indirectly impairs physical function through mechanisms such as reduced cardiac output, skeletal muscle wasting, and systemic inflammation.

Self-management, which is the ability of patients to actively participate in managing their condition via symptom monitoring, medication adherence, dietary compliance, and psychological adaptation, is a pivotal component of chronic HF care. Effective self-management has been increasingly recognized for its potential to influence disease trajectory and improve outcomes.^[4,5]^ The chronic care model provides the theoretical basis for this approach. This model emphasizes patient empowerment and proactive behavior, which help stabilize health status and prevent acute decompensation. However, it remains unclear which aspects of self-management have the greatest impact on long-term functional independence.

Therefore, this study aimed to investigate how key clinical features (such as comorbidities and biomarkers) and self-management domains together influence ADL outcomes over the 12 months following hospitalization for HF. We hypothesized the following: First, specific comorbidities, such as diabetes and valvular heart disease (VHD), along with elevated natriuretic peptide levels, would independently predict decreased ADL outcomes. Second, proficiency in self-management, especially in symptom recognition and response, would strongly predict ADL preservation. Third, these associations would differ significantly between patients with heart failure with reduced ejection fraction (HFrEF) and those with heart failure with preserved ejection fraction (HFpEF). By identifying these modifiable and non-modifiable predictors, this study sought to inform targeted interventions aimed at preserving functional capacity in this vulnerable population.

## 2. Materials and methods

### 2.1. Study design

**Figure 1.**
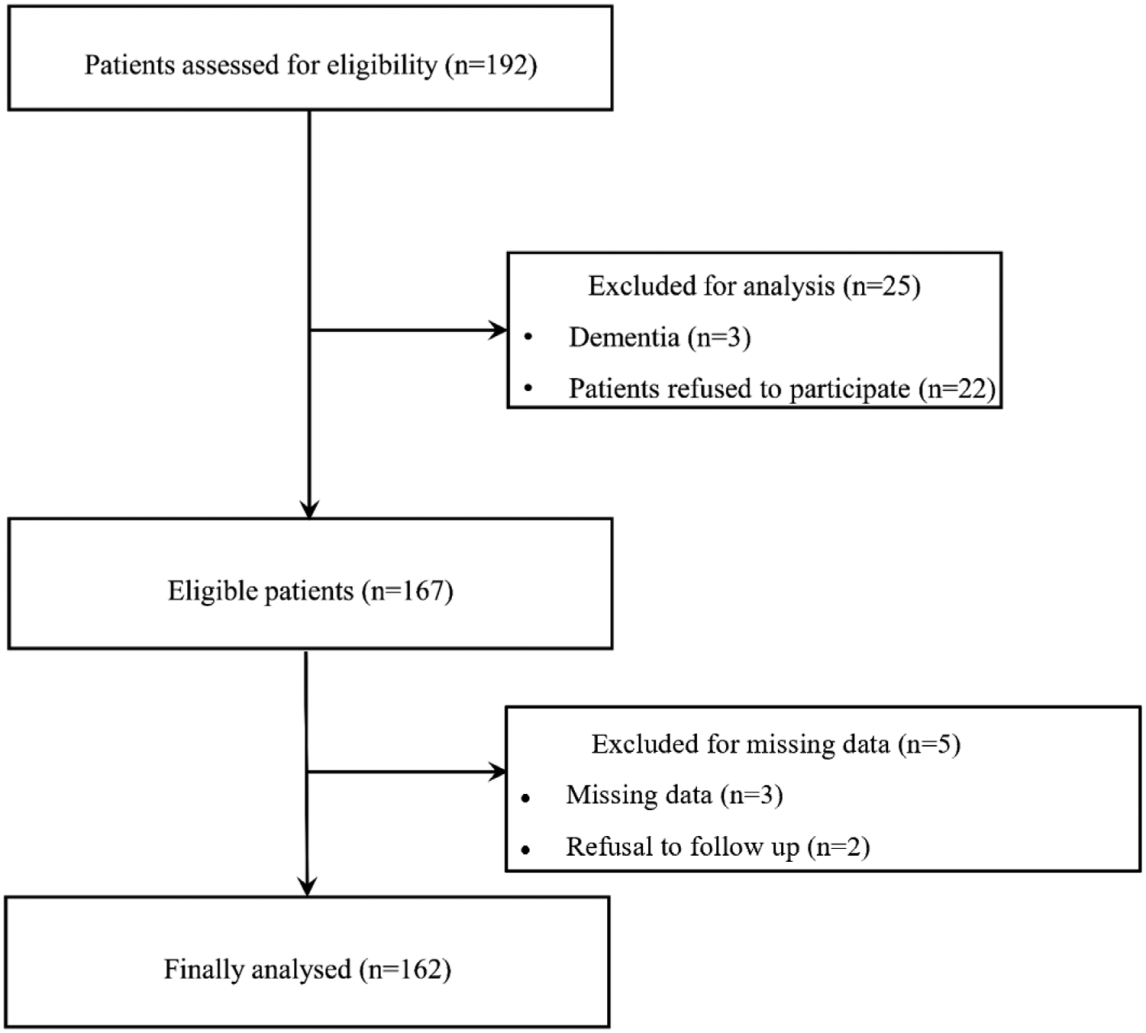
Flow chart of patient enrollment and follow-up.

This prospective cohort study enrolled adults hospitalized for chronic HF between March 2022 and December 2023 at a large general hospital in northern China. For follow-up, standardized assessments were performed at discharge and at 1, 3, 6, 9, and 12 months post-discharge (Fig. 1). Based on the ADL scores at the final follow-up, the patients were stratified into a high-ADL group (ADL score = 100) or a low-ADL group (ADL score < 100).

### 2.2. Data collection

Regarding ADL assessment, patients were stratified into high-ADL or low-ADL groups based on the attainment of 100 points on the Barthel Index^[6]^ at 12 months post-discharge. Second, we conducted analyses to investigate whether the 6-month ADL status and other time-varying factors could serve as early indicators of the 12-month outcomes.

The baseline variables in this study included demographics (age, sex, and educational level). The comorbidities assessed were coronary heart disease (CHD), chronic kidney disease (CKD), COPD, hypertension, VHD, atrial fibrillation, diabetes, and post-MI HF (MIHF). Cardiac function variables included left ventricular ejection fraction (LVEF; %) and B-type natriuretic peptide (BNP; pg/mL).

Self-management was assessed by measuring the Barthel Index across the following domains: psychological and adaptive management (%), drug management (%), dietary guidance (%), symptom management (%), and overall self-management ability (%). Other assessments included the nutrition and MUSIC risk scores.

### 2.3. Participants

#### 2.3.1. Eligibility criteria

This study included adult patients (age ≥ 18 years) hospitalized for chronic HF, which was diagnosed based on cardiac structural or functional abnormalities, including at least one of the following: impaired LVEF (≤50%), structural heart disease (e.g., left ventricular hypertrophy or significant left atrial enlargement), and diastolic dysfunction; laboratory evidence of HF, defined as elevated natriuretic peptide levels; and clinical context, including a documented history of chronic HF.

#### 2.3.2. Exclusion criteria

Patients were excluded if they met any of the following conditions: the presence of a noncardiac life-limiting illness (e.g., advanced malignancy or end-stage liver disease) with an expected survival prognosis deemed insufficient to complete the follow-up study, documented cognitive impairment precluding the patient’s ability to comprehend the study protocol or complete the required assessments and questionnaires, and any other reason that would prevent the completion of the standardized 12-month follow-up (e.g., residing in a remote geographic location or another factor associated with a high risk of loss to follow-up). Patients with missing data were also excluded from the final analysis.

#### 2.3.3. Sources of participants

All potential participants were admitted to the Tianjin Third Central Hospital during the specified enrollment period.

#### 2.3.4. Methods of selection

Eligible subjects were recruited through consecutive enrollment and systematic screening of eligible hospitalized patients with HF. Before the study was initiated, all the participants provided written informed consent. The study protocol was approved by the Institutional Review Board of Tianjin Third Central Hospital (Approval ID: 2022-003-04). Furthermore, in accordance with the predefined analysis plan, patients who died during follow-up were excluded from the primary analysis to accurately evaluate the determinants of long-term functional status among survivors.

### 2.4. Variables and measurement outcome variables

ADL status at 12 months post-discharge was the primary binary outcome. Patients were classified into the low-ADL group if their Barthel Index score was <100 and into the high-ADL group if their Barthel Index score was 100.

ADL was assessed using the Chinese version of the Barthel Index, a validated 10-item scale that assesses independence in basic self-care and mobility. The total scores range from 0 (complete dependence) to 100 (complete independence). Assessments were conducted at baseline and at 3, 6, and 12 months through structured interviews conducted by trained research nurses. To ensure consistency, all assessors participated in a standardized training session, and the inter-rater reliability was verified before the study began. The interviews focused on the patient’s actual performance during the preceding 24 to 48 hours, with additional clarification sought from primary caregivers when necessary.

#### 2.4.1. Exposures and predictors of interest

*Primary exposures included key predictors*: comorbidities, the presence of diabetes mellitus, and VHD documented in medical records; self-management domains: 4 core domains were assessed: psychological and adaptive management, drug management, dietary guidance, and symptom management. Each domain was assessed by using a continuous percentage score.

*Potential confounders*: Potential confounders adjusted in the multivariable models included age, sex, and BNP level.

#### 2.4.2. Effect modifier

The LVEF was assessed as a key effect modifier. The patients were stratified into 2 subgroups for analysis: HFpEF (LVEF ≥ 50%) and HFrEF (LVEF < 50%).

### 2.5. Data sources and assessment methods

#### 2.5.1. ADL assessment

The Barthel Index was used to assess ADL at discharge and at 1, 3, 6, 9, and 12 months post-discharge. The same instrument and assessment protocol were applied identically to all participants, ensuring comparability between the low- and high-ADL groups. The primary outcome was dependency in ADL, defined as a Barthel Index score of <100, assessed at the 12-month follow-up. The ADL status at the 6-month follow-up was assessed as a secondary/exploratory outcome measure for identifying early predictive signals.

#### 2.5.2. Clinical variables and comorbidities

Data on demographics (age, sex, education) and comorbidities, such as CHD, hypertension, diabetes, and VHD, were extracted from electronic medical records at baseline. The diagnostic criteria were based on established clinical guidelines and physician diagnoses.

#### 2.5.3. Biomarkers

LVEF was quantitatively measured using transthoracic echocardiography. BNP levels were determined using standard laboratory blood tests.

#### 2.5.4. Self-management assessment

Self-management abilities were assessed using a standardized instrument that generated percentage scores for each domain. All the participants completed this assessment, ensuring comparability across the groups.

### 2.6. Bias

#### 2.6.1. Selection bias

A prospective cohort design was used, enrolling patients during a defined period to minimize selection bias.

#### 2.6.2. Measurement bias

Standardized and validated tools (e.g., the Barthel Index and echocardiography) were used for key outcomes and exposures. Data collectors were trained to ensure consistent application of these tools across all patients.

#### 2.6.3. Confounding

Major known confounders (including age, sex, and BNP levels) and other relevant self-management domains were adjusted for in the multivariable logistic regression analysis to isolate the independent effect of the primary predictors.

#### 2.6.4. Loss to follow-up

To mitigate bias from differential loss to follow-up, a primary analysis was performed on patients who completed the 12-month assessment. Patients who died during follow-up were excluded from the primary analysis according to the study protocol, as the focus was on functional status among survivors.

### 2.7. Study size

The sample size of this study was determined based on the total number of eligible patients recruited during a 22-month inclusion period. A post hoc efficacy assessment showed 66 outcome events (low ADL) and approximately 7 predictor variables. The event-to-variable ratio was >9, which conforms to the commonly accepted rule for sample-size estimation. This indicates that the study has acceptable statistical power to detect predictors with moderate to strong effects.

Patients were categorized based on the median symptom management scale score at the time of enrollment. Subsequently, all patients were classified into the high and low symptom management score groups.

### 2.8. Statistical analysis

Continuous variables are presented as the mean ± standard deviation if normally distributed or as the median and interquartile range if non-normally distributed. Categorical variables are presented as frequencies and percentages. The primary grouping (low ADL vs high ADL) was based on clinical data. Primary analysis: univariate and multivariable binary logistic regression models were used to identify predictors of the outcome (low ADL). The multivariable model was specifically constructed to control for confounding factors by including age, sex, BNP, and the key self-management domains (psychological, drug, and dietary) as covariates. The measure of association used in this study was the odds ratio (OR) with a 95% confidence interval (CI). Subgroup analysis: prespecified subgroup analyses were conducted by stratifying the cohort by LVEF (HFpEF vs HFrEF). Logistic regression models were run separately within each subgroup to examine whether the associations between primary predictors (diabetes, symptom management, and valvular disease) and low ADL differed between these pathophysiologically distinct patient groups. Missing data were deleted during the statistical analysis. All statistical analyses were performed using SPSS version 27.0 (IBM Corp.), and a *P*-value < .05 was considered statistically significant. Sensitivity analyses were conducted using the elimination test one by one.

## 3. Results

### 3.1. Baseline characteristics

In total, 192 patients were assessed for eligibility. Among them, 25 were ineligible, 3 had dementia, and 22 refused to participate. Five patients were excluded because of missing data 3 months after enrollment. Ultimately, 162 patients were included in this analysis. The reasons for participant withdrawal and the amount of missing data are shown in Figure 1.

### 3.2. Descriptive data

This study included 162 hospitalized patients with chronic HF. All the patients completed 12 months of follow-up. Based on the Barthel Index scores at 12 months, which assess ADL, participants were stratified into a low-ADL group (ADL < 100, n = 66) and a high-ADL group (ADL = 100, n = 96). The 2 groups did not differ significantly in demographic characteristics: mean age was 71.8 ± 9.7 versus 72.1 ± 11 years (*P* = .86), the proportion of males was 62% versus 51% (*P* = .16), and the educational level distribution showed no significant difference (*P *= .93). The prevalence rates of VHD (12% vs 3%, *P* = .025) and diabetes (29% vs 16%, *P* = .043) were significantly higher in the low-ADL group, and the median BNP level was higher (474.9 vs 398.0 pg/mL, *P* = .039) in the same group. All specific self-management domain scores were significantly lower in the low-ADL group for psychological and adaptive management (70% vs 80%, *P* = .005). Although the scores for drug management (70% vs 80%, *P *= .005), dietary guidance (70% vs 80%, *P* = .028), and symptom management (60% vs 70%, *P* < .001) were reported as identical percentages, the statistical significance suggests differences in the distribution or other aspects of these domains. Other comorbidities, including CHD, CKD, COPD, hypertension, atrial fibrillation, and HF after myocardial infarction, did not differ significantly between the groups (Table 1). Potential confounders, such as age, sex, BNP level, and self-management domains (psychological, drug, and dietary management), were adjusted in the multivariable analyses.

**Table 1:** Baseline clinical features of patients with heart failure 12 months post-discharge.

Categories	Low ADL n = 66	High ADL n = 96	*P* value
Age, yr	71.8 ± 9.7	72.1 ± 11	.86
Sex, male, n (%)	41 (62)	49 (51)	.16
Education level			.93
Primary school, n (%)	18 (27)	23 (24)	
Junior high school, n (%)	28 (42)	43 (45)	
High school, n (%)	14 (21)	19 (20)	
Junior college, n (%)	6 (9)	11 (11)	
CHD, n (%)	52 (79)	69 (72)	.32
CKD, n (%)	3 (5)	3 (3)	.64
COPD, n (%)	10 (15)	15 (16)	.93
Hypertension, n (%)	28 (42)	44 (46)	.67
Valvular heart disease, n (%)	8 (12)	3 (3)	.025
Atrial fibrillation, n (%)	15 (23)	27 (28)	.44
Diabetes, n (%)	19 (29)	15 (16)	.043
MIHF, n (%)	12 (18)	27 (28)	.15
LVEF (%)	50 (40, 60)	50 (40, 60)	.710
BNP (pg/mL)	474.9 (207.0, 899.9)	398.0 (141.0, 576.7)	.039
Psychological and adaptive management, (%)	70 (60, 80)	80 (70, 90)	.005
Anxiety and depression scores	6.5 (2.0, 14.0)	6.0 (3.0, 10.0)	.73
Drug management, (%)	80 (70, 80)	80 (80, 90)	.005
Dietary guidance, (%)	80 (60, 90)	80 (70, 100)	.028
Symptom management, (%)	60 (50, 70)	60 (60, 80)	<.001
Self-management ability	70.0 (60.0, 90.0)	72.5 (60.0, 90.0)	.15
Nutrition scores	1.0 (0.0, 1.0)	0.0 (0.0, 1.0)	.31
MUSIC score	9.5 (3.0, 14.0)	7.0 (3.0, 14.0)	.31

ADL = activities of daily living, BNP = brain natriuretic peptide, CHD = coronary heart disease, CKD = chronic kidney disease, COPD = chronic obstructive pulmonary disease, LVEF = left ventricular ejection fraction, MIHF = heart failure after myocardial infarction.

There were no missing data for baseline variables, and all analyses were based on complete cases. Patient enrollment and follow-up processes are shown in Figure 1. This 12-month prospective cohort study calculated the follow-up time from discharge, with standardized assessments conducted at discharge and at 1, 3, 6, 9, and 12 months thereafter. The mean follow-up interval was 12 months, and the total follow-up interval was 162 person-years, with an average of 12 mo/patient.

### 3.3. Outcome data

**Figure 2.**
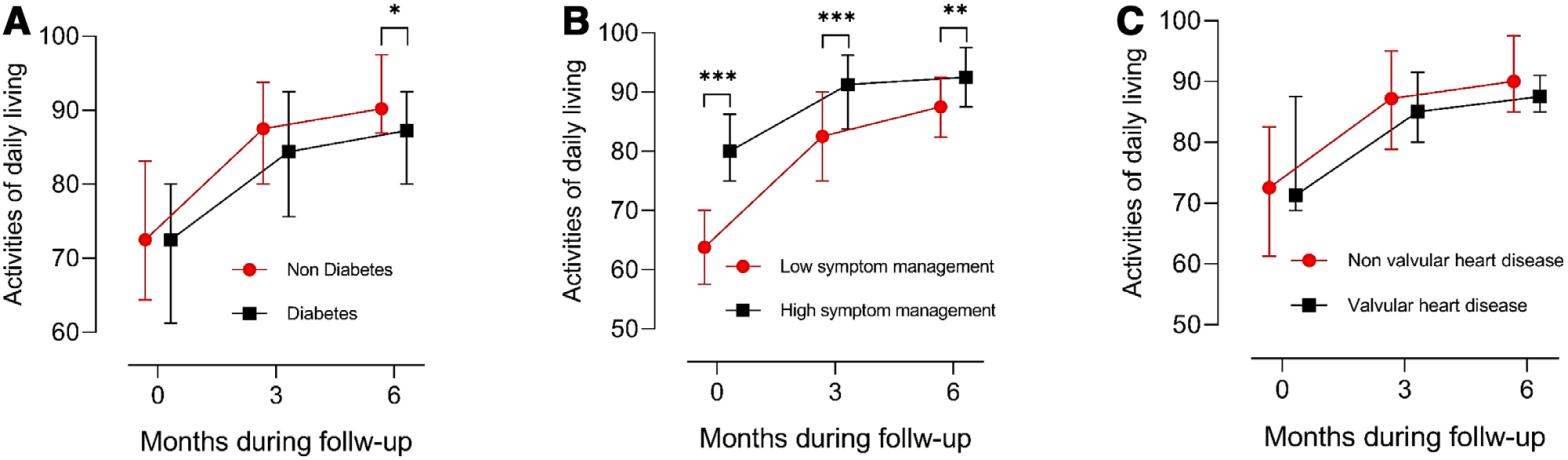
ADL scores at 6 months post-discharge in patients with heart failure: a subgroup analysis based on diabetes status, symptom management level, and valvular heart disease. (A) Effect of diabetes diagnosis on ADL scores (B) Effect of symptom management level (score of 100 vs. <100) on ADL scores (100: high symptom management level; <100: low symptom management level) (C) Effect of valvular heart disease diagnosis on ADL scores (*P < .05, **P < .01, ***P < .001).

In an exploratory analysis of the 6-month data to identify potential early predictors, we fitted a separate logistic regression model using ADL status at 6 months as the dependent variable. The ADL trajectory graphs (Fig. 2) showed significant divergence in ADL scores throughout the follow-up period. In particular, divergence in ADL scores occurred between patient groups defined by the key predictors. Patients with diabetes mellitus consistently exhibited lower ADL scores throughout the follow-up period than those without diabetes (Fig. 2A; *P *< .05). The high symptom management group maintained significantly higher ADL scores than the low symptom management group over time (Fig. 2B; *P *< .001). Patients with VHD showed significantly decreased ADL trajectories compared with those without VHD (Fig. 2C ; *P *< .01).

Logistic regression analysis identified the predictors of low ADL at 12 months post-discharge (Table 2). In the unadjusted model, diabetes (OR, 0.46; 95% CI, 0.21–0.99; *P* = .046), VHD (OR, 0.23; 95% CI, 0.06–0.92; *P* = .037), and symptom management (log-transformed OR, 12.71; 95% CI, 3.38–47.74; *P* < .001) were significant predictors. The multivariable-adjusted model, which controlled for potential confounders known to be associated with ADL outcomes, included age, sex, BNP level, psychological and adaptive management, drug management, and dietary guidance as covariates. After adjustment, diabetes (adjusted odds ratio [aOR], 0.33; 95% CI, 0.14–0.80; *P* = .014) and VHD (aOR, 0.19; 95% CI, 0.04–0.80; *P* = .024) remained independent predictors associated with decreased odds of low-ADL impairment, while symptom management, log-transformed for analysis, remained the strongest protective factor (aOR, 6.26; 95% CI, 1.44–27.19; *P *= .014).

**Table 2:** Logistic regression analysis of factors associated with activities of daily living at 12 months post-discharge.

	Crude model		Adjusted model	
	OR and 95% CI	*P* value	OR and 95% CI	*P* value
MIHF	1.76 (0.82–3.79)	.149	2.09 (0.85–5.10)	.106
Diabetes	0.46 (0.21–0.99)	.046	0.33 (0.14–0.80)	.014
Symptom management (Log)	12.71 (3.38–47.74)	<.001	6.26 (1.44–27.19)	.014
CHD	0.69 (0.33–1.44)	.321	0.76 (0.34–1.69)	.497
Valvular heart disease	0.23 (0.06–0.92)	.037	0.19 (0.04–0.80)	.024

The adjusted model included age, gender, BNP, psychological and adaptive management, drug management, and dietary guidance.

ADL = activities of daily living, BNP = brain natriuretic peptide, CHD = coronary heart disease, CI = confidence interval, MIHF = heart failure after myocardial infarction, OR = odds ratio.

### 3.4. Category boundaries for continuous variables

ADL grouping was based on the Barthel Index. LVEF was used for stratified analysis and was categorized as HFrEF (LVEF < 50%) and HFpEF (LVEF ≥ 50%). Self-management domains were expressed as percentages, and symptom management was log-transformed in regression analyses to improve the distribution.

### 3.5. Other analyses

**Figure 3.**
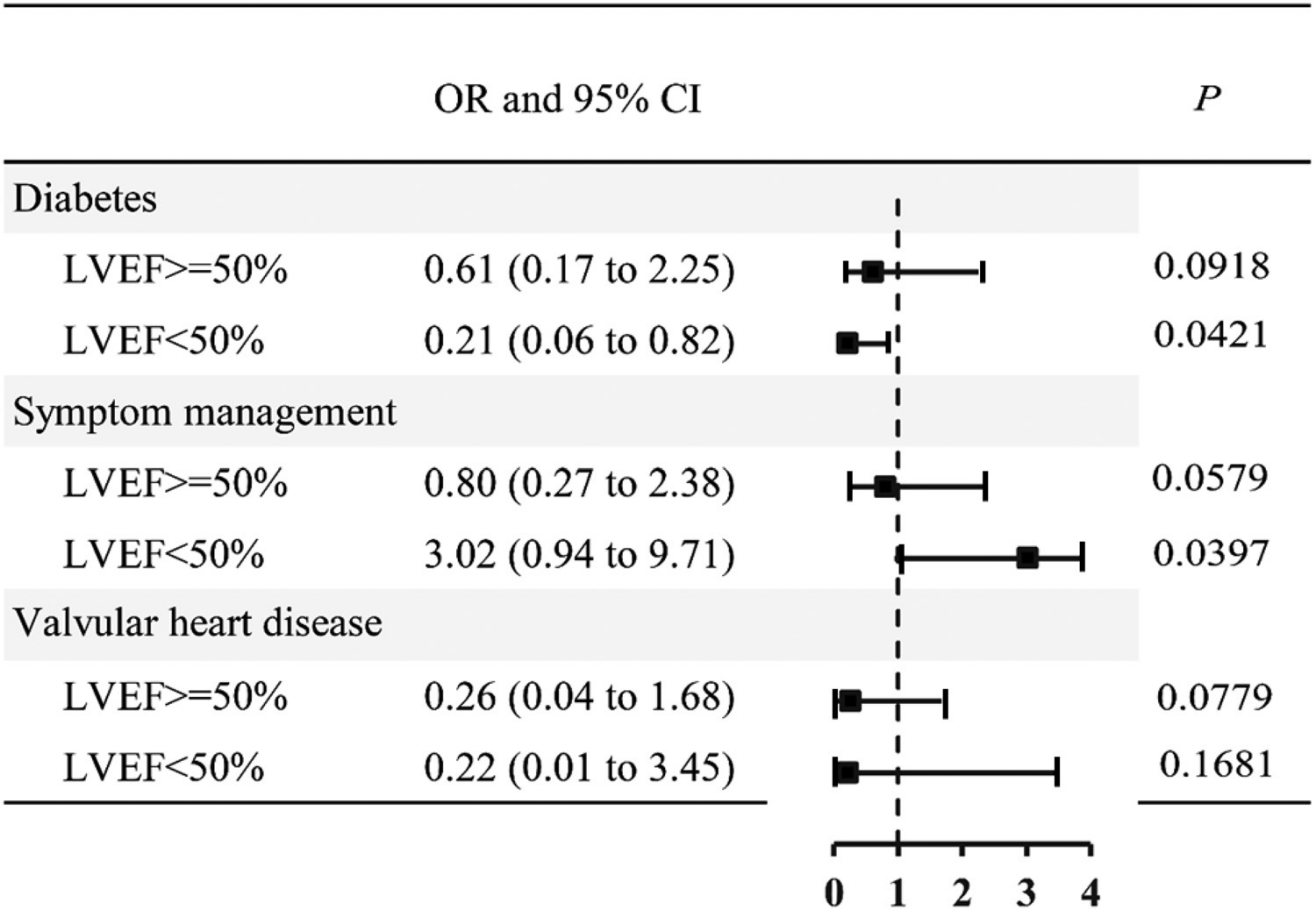
Activities of daily living scores at 12 months post-discharge stratified by left ventricular ejection fraction. CI = confidence interval, LVEF = left ventricular ejection fraction, OR = odds ratio.

ADL at 12 months post-discharge stratified by LVEF (Fig. 3): The impact of predictors differed significantly between patients with HFpEF (LVEF ≥ 50%) and those with HFrEF (LVEF < 50%), and diabetes had a significantly stronger negative impact on ADL in patients with HFrEF (OR, 0.21; 95% CI, 0.06–0.82) than in those with HFpEF (OR, 0.61; 95% CI, 0.17–2.25). Symptom management showed a strong trend toward being a more potent protective factor in patients with HFrEF (OR, 3.02; 95% CI, 0.94–9.71) than in those with HFpEF (OR, 0.80; 95% CI, 0.27–2.38), although the CI in HFrEF crossed 1. Regarding VHD, effect sizes did not differ significantly between the 2 groups (HFpEF OR 0.26, HFrEF OR 0.22); however, it is important to note that the CIs for some estimates in this analysis were wide, reflecting greater uncertainty, likely due to the small sample size in this interim assessment and its exploratory nature.

**Figure 4.**
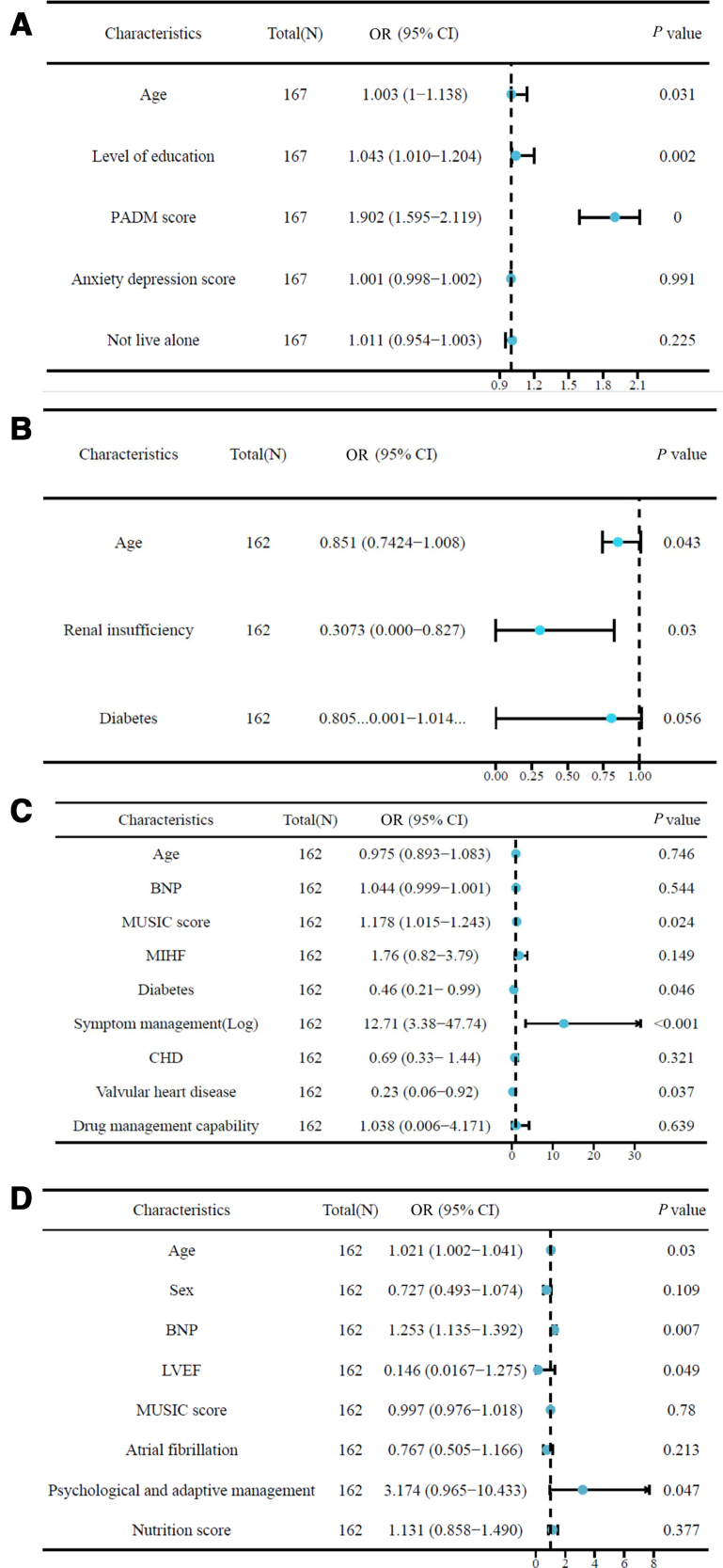
Forest plots from multivariable logistic regression analyses of factors associated with ADL at different follow-up time points: (A) in-hospital period, (B) 3 months post-discharge, (C) 6 months post-discharge, and (D) 12 months post-discharge. ADL = activities of daily living, CI = confidence interval, OR = odds ratio, PADM = psychological and adaptive management.

We analyzed the independent influencing factors of ADL in patients with different follow-up durations (Fig. 4). During the in-hospital period, age (OR, 1.003; 95% CI, 1.000–1.138; *P* = .031), education level (OR, 1.043; 95% CI, 1.010–1.204; *P* = .002), and psychological and adaptive management (PADM) scores (OR, 1.902; 95% CI, 1.595–2.119; *P* < .001) were significantly associated with ADL scores (Fig. 4A). At the 3-month follow-up, age (OR, 0.851; 95% CI, 0.742–1.008; *P* = .043) and CKD (OR, 0.307; 95% CI, 0.000–0.827; *P* = .03) emerged as significant factors, whereas diabetes showed a trend toward statistical significance (*P *= .056; Fig. 4B). At the 6-month follow-up, a higher MUSIC score (OR, 1.178; 95% CI, 1.015–1.243; *P* = .024), presence of diabetes (OR, 0.46; 95% CI, 0.21–0.99; *P* = .046), symptom management (log-transformed; OR, 12.71; 95% CI, 3.38–47.74; *P* < .001), and VHD (OR, 0.23; 95% CI, 0.06–0.92; *P* = .037) were identified as significant predictors of ADL outcomes (Fig. 4C). At the 12-month follow-up, the factors significantly associated with ADL outcomes included age (OR, 1.021; 95% CI, 1.002–1.041; *P* = .03), BNP level (OR, 1.253; 95% CI, 1.135–1.392; *P* = .007), LVEF (OR, 0.146; 95% CI, 0.0167–1.275; *P* = .049), and PADM (OR, 3.174; 95% CI, 0.965–10.433; *P* = .047; Fig. 4D).

## 4. Discussion

This study identified diabetes, VHD, and poor self-management of symptoms as key independent predictors of long-term ADL impairment in patients with HF. Additionally, the impact of these factors, particularly diabetes, may vary depending on the HF type (HFrEF vs HFpEF).

The robust association between diabetes and poor long-term ADL, particularly pronounced in patients with HFrEF, emphasizes the deleterious impact of metabolic comorbidities on functional capacity in HF.^[6]^ This aligns with evidence linking diabetes to skeletal muscle wasting, inflammation, and endothelial dysfunction in HF.^[7,8]^ Our findings extend prior work by demonstrating a differential effect by HF type: diabetes exerted a significantly stronger negative effect in HFrEF, suggesting distinct pathophysiological mechanisms such as exacerbated myocardial energy deficits and mitochondrial dysfunction.^[9]^

Similarly, VHD independently predicted ADL deterioration, reinforcing the role of structural cardiac abnormalities in limiting physical capacity.^[9,10]^ These pathologies progressively limit patients’ physical activity capacity through reduced cardiac output and elevated filling pressures.^[11]^ A 2023 meta-analysis emphasized that patients with HF already exhibit markedly reduced daily physical activity, with age-related declines significantly accelerated by comorbid conditions.^[12]^ Our findings are consistent with this observation.

The most clinically significant finding is the protective role of symptom management against functional decline, which aligns with several recent interventional studies demonstrating the multidimensional benefits of optimized self-management.^[13,14]^ Our results extend these findings by demonstrating that effective symptom management produces not only short-term clinical improvements but also long-term protective effects against functional decline. At 12 months, adaptive psychological management was a major protective factor in ADL maintenance. This finding resonates with recent research on the bidirectional relationship between psychological status and HF outcomes.^[15,16]^

Longitudinal analysis revealed that predictors of ADL changed over time: demographic factors dominated during hospitalization, whereas disease-specific and self-management factors gained importance post-discharge. These changes suggest that distinct intervention priorities may be appropriate for the different phases of the HF journey.^[17]^ This aligns with the growing recognition that functional capacity and independence are crucial determinants of quality of life, particularly in older adults.^[18,19]^ Our finding that patients with low ADL had significantly higher BNP levels reinforces the connection between neurohormonal activation and functional impairment.^[20]^ Stratification by LVEF highlighted phenotype-specific differences. Diabetes had a stronger negative impact in HFrEF, whereas symptom management showed a trend toward greater protection in this subgroup, possibly due to more predictable symptom patterns.^[21–23]^ If confirmed, these distinctions could inform phenotype-specific self-management interventions. The unequal sample sizes between the low- and high-ADL groups may have introduced some instability in the regression estimates, specifically in subgroup analyses, and should be considered when interpreting the results.

The strong protective effect of symptom managementunderscores the importance of comprehensive self-management education.^[24]^ Recent studies implementing psychological interventions support this approach.^[25,26]^ Current evidence indicates that structured exercise, particularly high-intensity interval training, improves metabolic efficiency and facilitates substantial cardiac adaptations.^[27,28]^ Our findings suggest that embedding exercise interventions within comprehensive self-management support maximizes their effectiveness.

These findings have important clinical implications. Comprehensive baseline evaluation should include screening for diabetes, VHD, and ADL impairment to identify high-risk patients. Aggressive comorbidity management (e.g., glycemic control, timely valve intervention) and structured self-management education – particularly symptom monitoring – may preserve functional independence. The 6-month post-discharge period appears to be a critical window for initiating multidisciplinary interventions.^[28]^

This study has several limitations. Although a complete case analysis was performed due to the low proportion of missing data (<3%), we acknowledge that this approach may introduce bias if the missingness is not completely at random. However, considering the small number of excluded cases, the effect on the overall findings is likely minimal. The single-center design and homogeneous Chinese cohort limit generalizability to other populations. Additionally, the imbalance between ADL groups and the use of complete-case analysis may introduce bias, though the impact is likely minimal given the low rate of missing data.

In conclusion, our study identified diabetes, VHD, and symptom management as the key predictors of ADL trajectories in patients with HF. Overall, these findings emphasize the importance of comprehensive assessments and individualized interventions that address both the clinical and behavioral determinants of functional outcomes. By integrating these insights into clinical practice and future research, we may better preserve functional independence and quality of life in the growing HF population.

## Acknowledgments

The authors gratefully acknowledge the technical support provided by the Department of Cardiology, Tianjin Third Central Hospital .

## Author contributions

**Conceptualization:** Meng Ning.

**Formal analysis:** Chong Zhang.

**Methodology:** Meng Ning, Chong Zhang.

**Supervision:** Chenguang Zheng.

**Validation:** Yingwu Liu.

**Writing – original draft:** Zhiyuan Li.
